# Placebo-Controlled Efficacy of Percutaneous Coronary Intervention for Focal and Diffuse Patterns of Stable Coronary Artery Disease

**DOI:** 10.1161/CIRCINTERVENTIONS.120.009891

**Published:** 2021-08-03

**Authors:** Christopher A. Rajkumar, Matthew Shun-Shin, Henry Seligman, Yousif Ahmad, Takayuki Warisawa, Christopher M. Cook, James P. Howard, Sashiananthan Ganesananthan, Laura Amarin, Caitlin Khan, Ayesha Ahmed, Alexandra Nowbar, Michael Foley, Ravi Assomull, Niall G. Keenan, Joban Sehmi, Thomas R. Keeble, John R. Davies, Kare H. Tang, Robert Gerber, Graham Cole, Peter O’Kane, Andrew S.P. Sharp, Ramzi Khamis, Gajen Kanaganayagam, Ricardo Petraco, Neil Ruparelia, Iqbal S. Malik, Sukhjinder Nijjer, Sayan Sen, Darrel P. Francis, Rasha Al-Lamee

**Affiliations:** 1 National Heart and Lung Institute, Imperial College London, United Kingdom (C.A.R., M.S.-S., H.S., Y.A., T.W., J.P.H., S.G., L.A., C.K., A.N., M.F., R.A., G.C., R.K., R.P., N.R., S.N., S.S., D.P.F., R.A.-L.).; 2 Imperial College Healthcare NHS Trust, London, United Kingdom (C.A.R., M.S.-S., H.S., J.P.H., A.N., M.F., R.A., G.C., R.K., G.K., R.P., N.R., I.S.M., S.N., S.S., D.P.F., R.A.-L.).; 3 Columbia University Medical Centre, New York–Presbyterian Hospital (Y.A.).; 4 St. Marianna University School of Medicine, Yokohama, Japan (T.W.).; 5 Essex Cardiothoracic Centre, Basildon, United Kingdom (C.M.C., T.R.K., J.R.D., K.H.T.).; 6 University of Sheffield, United Kingdom (A.A.).; 7 West Hertfordshire Hospitals NHS Trust, Watford, United Kingdom (N.G.K., J.S.).; 8 Anglia Ruskin School of Medicine, Chelmsford, Essex, United Kingdom (T.R.K., J.R.D.).; 9 East Sussex Healthcare NHS Trust, Hastings, United Kingdom (R.G.).; 10 Royal Bournemouth and Christchurch NHS Trust, Bournemouth, United Kingdom (P.O.).; 11 University Hospital of Wales, Cardiff, United Kingdom (A.S.P.S.).; 12 University of Exeter, United Kingdom (A.S.P.S.).

**Keywords:** angina pectoris, coronary artery disease, hemodynamics, ischemia, percutaneous coronary intervention

## Abstract

**Background:**

Physiological assessment with pressure wire pullback can characterize coronary artery disease (CAD) with a focal or diffuse pattern. However, the clinical relevance of this distinction is unknown. We use data from the ORBITA trial (Objective Randomised Blinded Investigation With Optimal Medical Therapy of Angioplasty in Stable Angina) to test if the pattern of CAD predicts the placebo-controlled efficacy of percutaneous coronary intervention (PCI) on stress echocardiography ischemia and symptom end points.

**Methods:**

One hundred sixty-four patients in ORBITA underwent blinded instantaneous wave-free ratio (iFR) pullback assessment before randomization. Focal disease was defined as a ≥0.03 iFR unit drop within 15 mm, rather than over a longer distance. Analyses were performed using regression modeling.

**Results:**

In the PCI arm (n=85), 48 were focal and 37 were diffuse. In the placebo arm (n=79), 35 were focal and 44 were diffuse. Focal stenoses were associated with significantly lower fractional flow reserve (FFR) and iFR values than diffusely diseased vessels (mean FFR and iFR, focal 0.60±0.15 and 0.65±0.24, diffuse 0.78±0.10 and 0.88±0.08, respectively, *P*<0.0001). With adjustment for this difference, PCI for focal stenoses resulted in significantly greater reduction in stress echo ischemia than PCI for diffuse disease (*P*<0.05). The effect of PCI on between-arm pre-randomization adjusted exercise time was 9.32 seconds (95% CI, −17.1 to 35.7 seconds; *P*=0.487). When stratified for pattern of disease, there was no detectable difference between focal and diffuse CAD (*P*interaction=0.700). PCI improved Seattle Angina Questionnaire angina frequency score and freedom from angina more than placebo (*P*=0.034; *P*=0.0035). However, there was no evidence of interaction between the physiological pattern of CAD and these effects (*P*interaction=0.436; *P*interaction=0.908).

**Conclusions:**

PCI achieved significantly greater reduction of stress echocardiography ischemia in focal compared with diffuse CAD. However, for symptom end points, no such difference was observed.

**Registration:**

URL: https://www.clinicaltrials.gov; Unique Identifier: NCT02062593.

## Abstract 2

Supplemental Digital Content is available in the text.

**What Is Known**

- Pressure wire pullback characterizes the physiological pattern of epicardial coronary artery disease as focal or diffuse.
- In the ORBITA trial (Objective Randomised Blinded Investigation With Optimal Medical Therapy of Angioplasty in Stable Angina), percutaneous coronary intervention was highly effective in relieving myocardial ischemia; yet in comparison to placebo, its benefits for symptomatic end points were smaller than expected.

**What the Study Adds**

- Focal stenoses are associated with significantly lower fractional flow reserve and instantaneous wave-free ratio values than diffusely diseased vessels.
- When this difference is adjusted for, percutaneous coronary intervention offers significantly greater reduction of stress echocardiography ischemia in focal rather than in diffuse pressure wire pullback patterns.
- However, despite this difference, stratifying patients according to the pattern of coronary artery disease (focal versus diffuse) does not seem to be an effective means of predicting placebo-controlled symptomatic benefit from percutaneous coronary intervention.

**See Editorial by Raharjo and Kunadian**

Clinical outcome trials which have supported the use of invasive coronary physiology apply diagnostic cut-points to dichotomize decision-making for revascularization in stable coronary artery disease (CAD).^1–3^ Fractional flow reserve (FFR) and the instantaneous wave-free ratio (iFR) assess the pressure gradient across a vessel, as an objective index of severity of CAD. However, atherosclerosis within a vessel is more complex than is described by a singular FFR or iFR value. Both focal and diffuse patterns of disease are well recognized; but their impact on the efficacy of revascularization is not well understood.

The pattern of CAD may be assessed anatomically (using quantitative coronary angiography [QCA] or intravascular imaging), or physiologically, using invasive pressure wire pullback. Physiological assessment characterizes the pattern of pressure loss longitudinally throughout a diseased coronary artery. iFR pullback offers lesion-specific assessment by quantifying the contribution of epicardial resistance apportioned to each vessel segment.^4,5^ By permitting assessment of the pressure gradient at any given point along the vessel, the pattern of disease can then be characterized as focal, diffuse, or a mixed pattern.^4^

It is plausible that percutaneous coronary intervention (PCI) of focal stenoses provides greater physiological and symptomatic benefits than PCI of diffusely diseased vessels. This may be for a variety of reasons, for example, it may result in shorter stented segments which are more easily optimized, or it may reflect a lower burden of atherosclerosis and microvascular disease. However, the efficacy of PCI for treatment of symptoms in focal and diffuse patterns of stable CAD has not been studied with placebo control.

The ORBITA trial (Objective Randomised Blinded Investigation With Optimal Medical Therapy of Angioplasty in Stable Angina) was the first placebo-controlled trial of PCI for stable CAD. It showed that PCI was effective in normalizing the anatomic and hemodynamic features of a coronary stenosis; yet when compared with placebo, the effects on exercise time and symptoms were smaller than expected.^6^

In a prespecified analysis from ORBITA, the association between pre-randomization FFR and iFR and the clinical end points was studied.^7^ As might be expected, progressively lower, more ischemic, pre-randomization FFR and iFR values were associated with greater improvements in stress echocardiography ischemia with placebo-controlled PCI. However, surprisingly, there was no association between pre-randomization FFR and iFR and the placebo-controlled benefit of PCI on exercise time, symptoms, or quality of life.

In this analysis, we use iFR pullback data and angiographic criteria to stratify the results of ORBITA to determine whether the placebo-controlled efficacy of PCI may instead be dependent on the pattern of CAD: focal or diffuse.

## Methods

### Study Design

The ORBITA trial was approved by the London-Central Research Ethics Committee; the design of the trial has been previously described.^6^ All subjects gave informed consent. The data, analytical methods, and study materials will not be made available to other researchers.

### Invasive Physiological Assessment

Patients underwent auditory isolation with over-the-ear headphones playing music. Invasive physiological assessments were completed with the primary operator, a consultant interventional cardiologist, blinded to their results as previously discussed.^7^ In brief, this was to ensure that patients with a clinically representative range of values for FFR and iFR were randomized so that the placebo-controlled efficacy of PCI could be investigated across a full range of FFR and iFR values to investigate the cut-points for improvement in angina.

Following iFR and FFR assessment and return of Pd/Pa ratio to baseline, a blinded manual iFR pullback manoeuvre was recorded under resting conditions. The target pullback speed was 1 mm/second to bring the pressure sensor back to the tip of the guiding catheter. A drift check was then recorded. Where the Pd/Pa ratio fell outside of the range 1.00±0.02, the wire was re-normalized and blinded iFR, FFR, and iFR pullback assessments repeated with a further drift check performed.

### Randomization Procedure

After completion of physiological assessment, pharmacotherapy was administered to a deep level of conscious sedation. Once confirmed, patients were randomized 1:1 to PCI or a placebo procedure with blinding techniques as previously described.^6^ In the PCI arm, iFR and FFR were remeasured after revascularization, with the operator blinded to the results. Patients and all subsequent medical caregivers remained blinded to treatment allocation until the end of the follow-up period.

### Study End Points and Follow-Up

Follow-up was performed at the end of a 6-week blinded period as previously described.^6^

### Blinded Analysis of iFR Pullback Data

To characterize the physiological pattern of disease, each iFR pullback trace was assessed twice by 6 interventional cardiologists (R.A.L., N.R., T.W., Y.A., C.M.C., H.S.) who were blinded to subject identifiers, treatment allocation, the coronary angiogram, each other’s opinion and their own first opinion.

Each assessor was asked to grade the pattern of disease as focal, diffuse, or mixed pattern of disease. We applied a previously published definition from the DEFINE-PCI (Physiological Assessment of Coronary Stenosis Following PCI) Study.^8^ In brief, focal disease was defined as ≥0.03 iFR unit drop within 15 mm, rather than over a longer distance.

### QCA Analysis of Lesion Length

To provide a separate, angiographic assessment of the pattern of CAD, 3 independent investigators (Y.A., C.K., and A.A.) performed QCA analysis of the target vessel in each subject. The lesion length was calculated in each case. Where tandem stenoses were present within the same vessel, the sum of the lesion lengths was taken. The investigators were blinded to each other’s opinion, the iFR pullback tracing and the randomization arm.

### Dobutamine Stress Echocardiography

Dobutamine stress echocardiography was performed twice in each patient, once before randomization and again at the end of the 6-week blinded follow-up period. The patient, sonographer, and physician performing the study were blinded to treatment allocation, as were 6 cardiac imaging consultants who reported each study.^9^

### Statistical Analysis

This stratified analysis of ORBITA consists of all patients randomized in the ORBITA trial with pre-randomization iFR pullback.

All analysis has been performed with isolated “focal” disease tested against a combined group of patients with “diffuse” or “mixed” disease indicated by iFR pullback. We chose this model of analysis to optimally test the hypothesis that PCI would offer its greatest benefit in those patients with an isolated focal stenoses by physiological criteria and no diffuse disease. To be classified as a “focal” disease pattern for analysis, the majority opinion (at least 4) from the first assessment by the 6 raters needed to be “focal”. All traces not meeting these criteria were considered to incorporate diffuse disease: these traces formed the “diffuse” category.

For the anatomic classification, lesion length, as calculated by QCA, was treated as a continuous variable and tested for its impact on the placebo controlled benefit of PCI on stress echo score and symptom end points.

The Seattle Angina Questionnaire (SAQ) scale for angina frequency was derived from individual patient responses in accordance with published guidelines.^10^ Freedom from angina was defined from the SAQ as previously described.^11^

Regression models were used to provide increased statistical power and test the interaction between pattern of CAD as assessed by iFR pullback and QCA calculated lesion length on the placebo-controlled benefit of PCI on the stated end points.^12^ A model was fitted for each end point. For exercise time, a least squares model was fitted, whereas a proportional odds ordinal logistic model was used for SAQ angina frequency, freedom from angina, and stress echo score. The latter model accommodates for possible floor and ceiling effects of angina frequency as an end point. The pre-randomization values were modeled with a restricted cubic spline with 3 knots (placed at 25%, 50%, and 75% of the data) with the presence of focal or diffuse disease allowed to interact with the randomization arm.^12,13^

All analyses which compared focal and diffuse categories were adjusted for the baseline physiology by including a term for the iFR or FFR (as specified in the results) in the model. This allowed us to test the association of the pattern, as opposed to the severity of CAD, on the stated end points.

The inter- and intraobserver agreement between 6 assessors’ grading of iFR pullback traces was calculated using Fleiss’ Kappa.

The Open Source statistical programming environment R was used for all statistical analyses.^14^ The package rms^15^ was used for regression modeling and ggplot2 for all graphs.^16^

## Results

The ORBITA trial randomized 200 patients to PCI (n=105) or a placebo procedure (n=95). iFR pullback traces were not available for all patients because iFR pullback technology was not available at every recruiting site from the start of the recruitment period. In addition, in 3 patients, the lesion could not be crossed with a pressure wire and in 1 patient the pressure wire resulted in intimal disruption requiring immediate PCI. An iFR pullback trace was therefore available for 164 patients, of which 85 were randomly allocated to the PCI arm and 79 to the placebo procedure.

### Patient Demographics

Patient demographics are shown in Table 1.

**Table 1.**
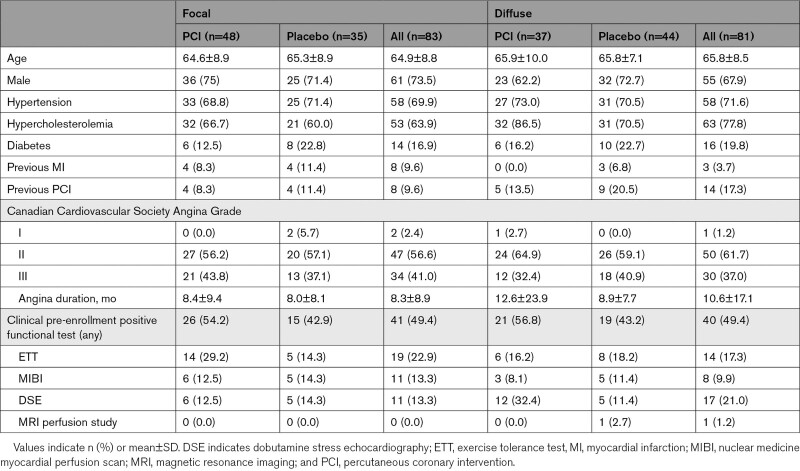
Patient Demographics at Enrollment

### Procedural Characteristics

Procedural characteristics are shown in Table 2.

**Table 2.**
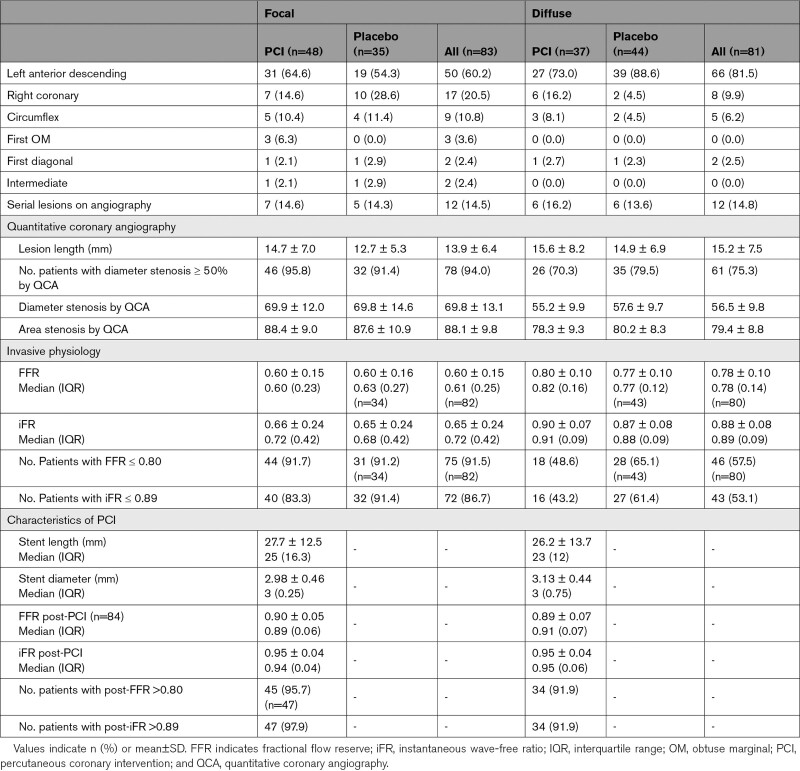
Procedural Characteristics

### Pattern of Disease as Assessed by iFR Pullback

One hundred sixty-four iFR pullback traces were assessed by 6 raters, resulting in 984 individual trace assessments from which a consensus was calculated. In the PCI arm (n=85), 48 (56%) were classified as focal and 37 (44%) were classified as diffuse. In the placebo arm (n=79), 35 (44%) were focal and 44 (56%) were diffuse.

### Study End Points

#### Relationship of Physiological Pattern of Disease and Change in Dobutamine Stress Echo Score With Placebo-Controlled PCI

Paired iFR pullback and dobutamine stress echocardiography data were available in 131 patients. When assessed with invasive physiology, focal stenoses were associated with significantly lower pre-randomization FFR and iFR values than diffusely diseased vessels (focal stenoses mean FFR and iFR 0.60±0.15 and 0.65±0.24, diffuse lesions mean FFR and iFR 0.78±0.10 and 0.88±0.08, respectively, *P*<0.0001; Table 2).

**Figure 1.**
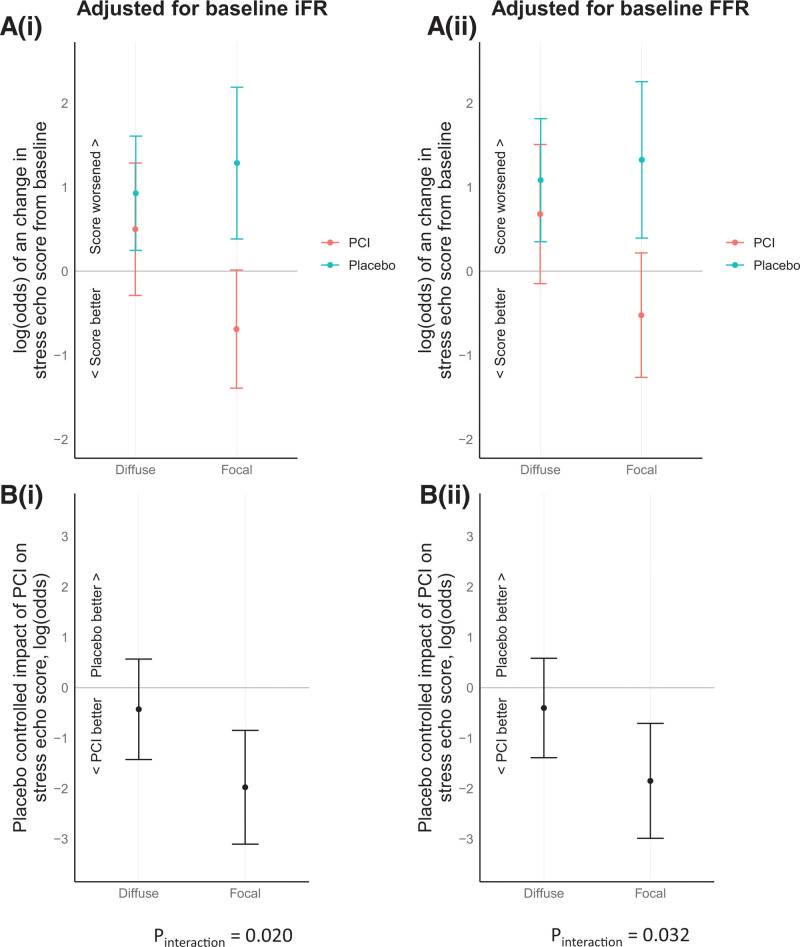
**Impact of placebo-controlled percutaneous coronary intervention (PCI) on dobutamine stress echocardiography (DSE) ischemia. A**, Impact of PCI and placebo on DSE ischemia according to physiological pattern of disease, adjusted for baseline instantaneous wave-free ratio (iFR) (**A(i**)) and baseline fractional flow reserve (FFR) (**A(ii**)). **B**, Placebo-controlled impact of PCI on DSE ischemia in diffuse and focal stenoses, adjusted for baseline iFR (**B(i**)) and baseline FFR (**B(ii**)).

Across the cohort, PCI resulted in significantly larger improvement in stress echocardiography documented ischemia in comparison to a placebo procedure, OR, 3.44 (95% CI, 1.83–6.47, *P*<0.0001). After adjustment for the difference in pre-randomization FFR and iFR values between focal and diffuse stenoses, PCI for focal stenoses offered significantly greater reduction in stress echo ischemia than PCI for diffuse disease. This effect was consistent when adjusted for baseline iFR (*P*=0.020) or baseline FFR (*P*=0.032) values (Figure 1).

#### Exercise Time

**Figure 2.**
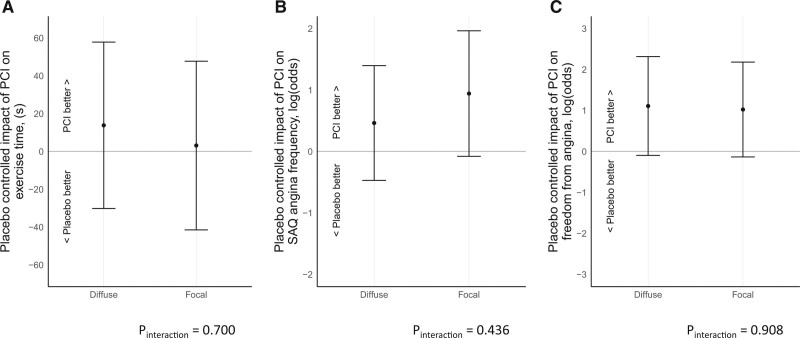
**Effect of physiological pattern of disease on the impact of placebo-controlled percutaneous coronary intervention (PCI) on symptom end points.** The association between pattern of coronary artery disease as assessed by instantaneous wave-free ratio (iFR)-pullback and the benefit of PCI over placebo for (**A**) exercise time, (**B**) Seattle Angina Questionnaire (SAQ) angina frequency, and (**C**) SAQ derived freedom from angina, after adjustment for baseline iFR values.

Paired exercise time data and iFR pullback assessments were available for 158 patients (84 in the PCI arm and 74 in the placebo arm). In this cohort, the estimated effect of PCI over placebo on exercise time using regression modeling was 9.32 seconds (95% CI, -17.1 to 35.7 seconds; *P*=0.487). For this relatively small effect, there was no detectable evidence of interaction between a focal disease pattern and the effect of PCI on exercise time increment after adjustment for baseline iFR (*P*interaction=0.700, Figure 2A) or baseline FFR (*P*interaction=0.615, Figure IA in the Data Supplement).

#### Seattle Angina Questionnaire Angina Frequency Score

Paired SAQ angina frequency data and iFR pullback were also available for 158 patients (84 in the PCI arm and 74 in the placebo arm). In this cohort, PCI significantly improved SAQ angina frequency score over a placebo procedure (odds ratio, 1.88 [95% CI, 1.05–3.37]; *P*=0.034). However, there was no statistically significant evidence of interaction between a focal disease pattern and the effect of PCI on angina frequency when adjusted for baseline iFR (*P*interaction=0.436, Figure 2B), or baseline FFR (*P*interaction=0.586, Figure IB in the Data Supplement).

#### Freedom From Angina

Within this cohort, PCI was more likely to result in patient-reported freedom from angina than placebo (odds ratio, 2.90 [95% CI, 1.42–5.92]; *P*=0.0035). However, there was no detectable evidence of interaction between the presence of a focal disease pattern and the effect of PCI on the likelihood of achieving freedom from angina after adjustment for baseline iFR (*P*interaction=0.908, Figure 2C) or FFR (*P*interaction=0.797, Figure IC in the Data Supplement).

#### Impact of Lesion Length as Assessed by QCA on Placebo-Controlled Efficacy of PCI

**Figure 3.**
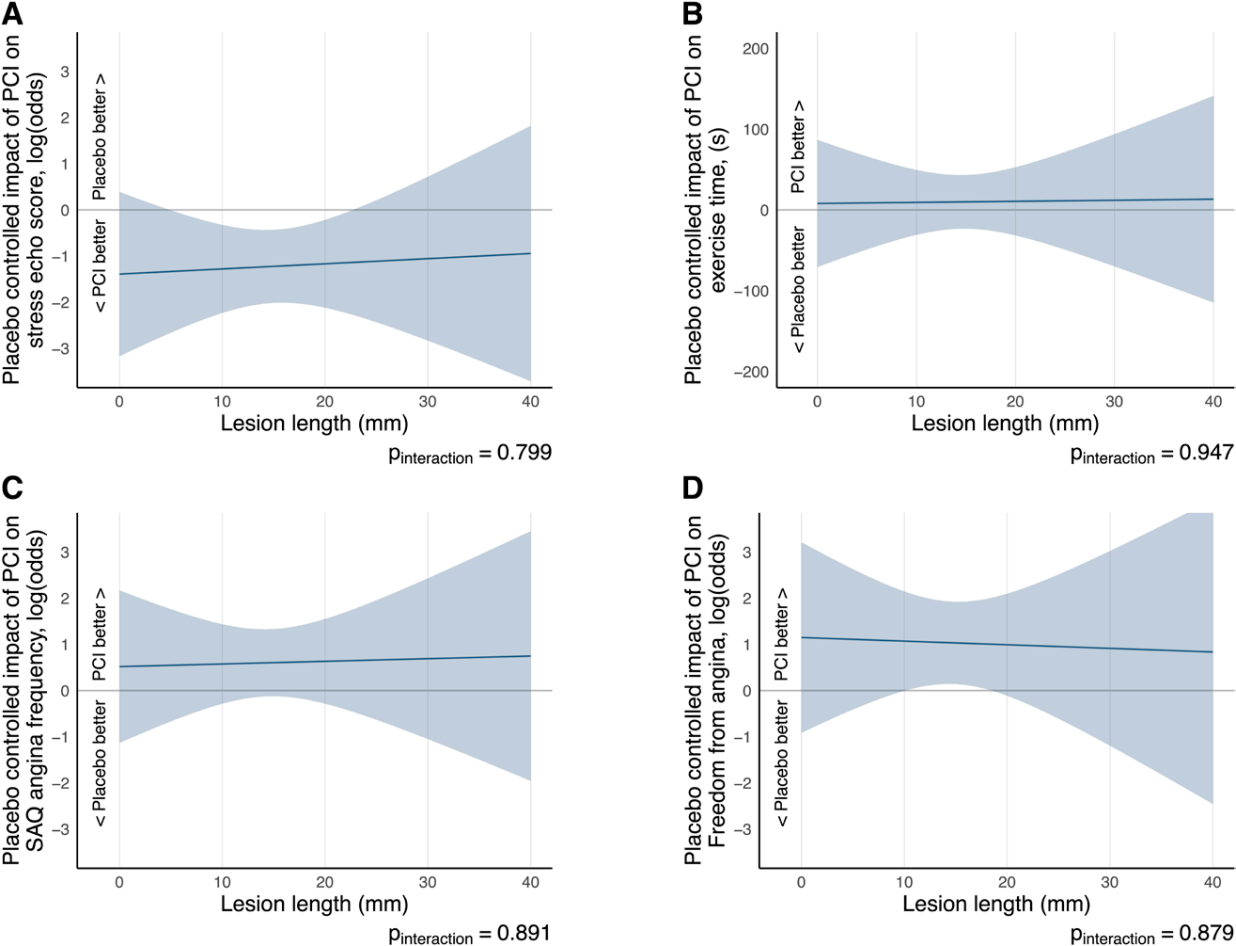
**Impact of quantitative coronary angiography lesion length on placebo-controlled efficacy of percutaneous coronary intervention (PCI) on dobutamine stress echocardiography and symptom end points.** The impact of lesion length on (**A**) stress echo score, (**B**) exercise time, (**C**) Seattle Angina Questionnaire (SAQ) angina frequency, and (**D**) SAQ derived freedom from angina.

Blinded QCA lesion length assessments were performed in 163 of the 164 patients (99.3%) included in this analysis. This provided an angiographic, as opposed to a physiological stratification of the pattern of disease. There was no significant impact of lesion length on the placebo-controlled impact of PCI on stress echo score (*P*interaction=0.799, Figure 3A). Furthermore, when lesion length was used as a predictor of the placebo-controlled impact of PCI on symptom end points, there was no significant effect on exercise time (*P*interaction=0.947, Figure 3B), SAQ angina frequency score (*P*interaction=0.891, Figure 3C) or freedom from angina (*P*interaction=0.879, Figure 3D).

#### Inter and Intraobserver Agreement in Assessment of Pattern of Disease by iFR Pullback and Dobutamine Stress Echocardiography Reporting

There was good interobserver agreement across 6 raters, each assessing 164 individual pullback traces (Fleiss’ Kappa 0.603). The intraobserver agreement between an individual assessor’s first and second assessments of pullback traces was also good (Fleiss’ Kappa 0.799).

The mean inter- and intraobserver absolute differences of the stress echocardiography score were 1.3 and 0.9 stress echocardiography units, respectively.

#### Sensitivity Analysis

Our sensitivity analysis restricted the eligible cohort to those with pre-randomization physiological values of FFR ≤0.80 (Table I in the Data Supplement), and iFR ≤0.89 (Table II in the Data Supplement).

## Discussion

This is the first placebo-controlled data to assess the efficacy of PCI stratified by the physiological pattern of CAD. Focal stenoses were associated with significantly lower pre-randomization FFR and iFR values than diffusely diseased vessels. After adjustment for the difference in absolute ischemia, we found that PCI for physiologically focal stenoses offered significantly greater improvements in stress echocardiography documented ischemia than PCI for physiologically diffuse disease.

However, no such relationship was observed for the symptom end points. Specifically, when adjusted for baseline iFR and FFR values, there was no detectable difference in the placebo-controlled increase in exercise time following PCI for physiologically focal compared with diffuse disease. Similarly, the physiological pattern of disease did not predict the placebo-controlled efficacy of PCI on angina frequency or freedom from angina.

Our second analysis, which stratified patients according to anatomic rather than physiological criteria, showed no association between QCA lesion length and the placebo-controlled impact of PCI on stress echocardiography ischemia or any symptom end point. Physiologically diffuse lesions were only marginally longer anatomically than physiologically focal stenoses. This likely reflects the weak correlation between visual assessment of the coronary angiogram and the invasive physiological pattern of disease when assessors are blinded to the physiological results.

Previous stratified analyses of ORBITA have tested the association between severity of pre-randomization ischemia and symptomatic improvement following PCI.^7,9^ Given that there was no detectable interaction between invasive physiology (FFR or iFR) and the efficacy of PCI on exercise time or symptoms,^7^ we performed the present analysis to test the hypothesis that PCI may be more effective in treating focal stenoses than diffusely diseased arteries.

The theory for this assumption is clear: optimal PCI in diffuse disease is more challenging because the magnitude of physiological benefit per unit of stented segment is diminished. Furthermore, residual disease, particularly in the distal vessel, may contribute to ischemia, but will not be amenable to PCI.

ORBITA has helped our understanding of the relationship between coronary stenosis, ischemia, and symptoms. We assume that restriction of epicardial blood flow caused by coronary stenoses, focal or diffuse, results in hemodynamic insufficiency and myocardial ischemia during stress. With sufficient ischemia, a wall motion abnormality may become evident. However, the mass of ischemic myocardium required to cause downstream symptomatic manifestations (angina) was previously unknown.

The design of ORBITA allowed the sequence of steps in this model to be tested. Invasive physiological assessments of ischemia (FFR and iFR) predicted the improvement in stress echo ischemia from PCI but not placebo-controlled symptomatic benefit.^7^ However, the Dobutamine stress echocardiography-stratified analysis of ORBITA showed that at stress echo scores of ≥1 (ie, ≥1 segment of hypokinesia), PCI resulted in a placebo-controlled reduction in patient-reported frequency of angina.^9^ Overall, this suggests that targeting ischemia testing further downstream, where abnormalities reflect the total burden of ischemic myocardium, allows identification of patients that may be most likely to benefit symptomatically from PCI. While high-precision invasive physiological measurements made upstream in the cascade are vessel-specific and provide an invaluable measurement of ischemia in the catheterization laboratory, they may be too sensitive to make predictions of symptomatic benefit from PCI. This may explain the absence of an interaction between iFR pullback assessments and the efficacy of PCI on symptomatic end points.

However, if we accept that ischemia is a continuum and move away from dichotomous cut-points, tools such as iFR pullback have a valuable place in the management of stable CAD. By preventing the loss of information content that occurs through dichotomization, these tools present a more complete assessment of vessel characteristics. They are also useful for optimization of the hemodynamic result of PCI. The DEFINE-PCI study performed blinded iFR pullback following angiographically successful PCI, to determine the cause of any residual ischemia.^8^ It found that 81.6% of patients with an iFR value <0.90 post-PCI had untreated focal stenoses. Randomized data are required to test the utility of pullback technology. To this end, we await the results of the international DEFINE-GPS trial which will address event end points.^17^ For symptom end points, blinded studies are required.

The recently reported ISCHEMIA trial showed prognostic clinical equipoise for an invasive versus conservative strategy in stable CAD.^18^ Symptom and quality of life improvement is now the main goal of revascularization in this setting. However, the results of this analysis once again show that the relationship between ischemia and symptoms is much more complex than we had hoped.

### Study Limitations

iFR pullback data were available for 164 of the 200 randomized ORBITA participants (82%). This sample size may limit the power of this analysis. iFR pullback, rather than FFR pullback was used in this analysis because the use of a hyperemic index such as FFR for longitudinal vessel analysis has been shown to be limited by hemodynamic cross-talk between serial stenoses in the same vessel.^19^ Resting indices such as iFR pullback appear to be less vulnerable to this phenomenon.^4,20^

Patients were selected for inclusion in the ORBITA trial on the basis of single vessel coronary disease, symptomatic angina, and the absence of severe left ventricular impairment or severe valvular disease. This is a very specific cohort chosen because it makes it straightforward to draw inferences about the relationship between lesion characteristics and the placebo-controlled effect of PCI. Moreover, it should be remembered that the majority of elective PCI is conducted for single vessel disease.^21^ It is unknown from the present data whether diffuse disease involving multiple vessels instead of only a single vessel would respond any differently to PCI.

The prevalence of diabetes was low across the cohort, which may be reflective of the fact that only patients with single vessel disease were eligible for enrollment. This is unlikely to have artificially reduced the symptomatic effect of PCI. Patients with multivessel disease will be eligible for inclusion in the ORBITA-2 trial which is currently enrolling.

Interestingly, in this substudy of the ORBITA trial, placebo-controlled PCI resulted in an improvement in SAQ angina frequency score. While there was a trend to this relationship in previously published reports^7,9^ this was not statistically significant. This reflects chance differences in the cohorts eligible for the analyses.

Operators were blinded to iFR pullback traces during the randomization procedure and therefore did not use this data to guide PCI. Angiography alone may underestimate the extent of diffuse disease resulting in shorter stent length. This is reflected in the procedural characteristics which show no difference in stent length between the focal and diffuse categories. The results of this analysis should be interpreted in this context; physiologically diffuse disease may have been underappreciated by the blinded operators who could only use angiography and intravascular imaging, at their discretion, to guide their procedure. Larger trials are needed to study whether PCI guided by unblinded pressure wire pullback and co-registration technology, in addition to intravascular imaging, can improve outcomes.

It could be argued that a favorable disease pattern in combination with a sufficiently low absolute iFR or FFR value may identify symptomatic responders to PCI. However, we have not further stratified diffuse and focal categories according to their absolute distal iFR value because further subdivision of groups is likely to be underpowered. The larger sample size offered by the ORBITA-2 trial may permit further stratification of results according to disease pattern, degree of ischemia and per-vessel analysis (left anterior descending, circumflex and right coronary artery).

In the absence of an accepted gold-standard criteria to define a focal stenosis, we applied the same definitions utilized by the physiology Core Laboratory at the Cardiovascular Research Foundation for the DEFINE-PCI study.^8^ The result of this analysis is therefore subject to the criteria that were applied for the identification of focal disease. Furthermore, pressure wire pullback was not automated and instead was performed manually. Differences in the speed of pullback may have made precise determination of a 15 mm distance, as specified in our definition of focal disease, more challenging. Contemporary co-registration technology was not available for the majority of the time period of the trial but may aid similar analyses in the future.

## Conclusions

In this analysis of ORBITA, stratified by the physiological pattern of disease, focal stenoses were associated with significantly lower pre-randomization FFR and iFR values than diffusely diseased vessels. With adjustment for this difference, placebo-controlled PCI for focal stenoses offered significantly greater reduction in stress echocardiography ischemia than PCI of diffusely diseased arteries. However, we did not observe any independent effect of the pattern of CAD on the placebo-controlled benefit of PCI on symptom end points. The absence of an interaction between physiological pattern of disease and symptom improvement from placebo-controlled PCI may reflect a weak association between invasive hemodynamic changes and symptom relief and requires further study.

## Sources of Funding

The ORBITA trial (Objective Randomised Blinded Investigation With Optimal Medical Therapy of Angioplasty in Stable Angina) was funded by grants from National Institute for Health Research (NIHR) Imperial Biomedical Research Centre, Foundation for Circulatory Health, and Imperial College Healthcare Charity. Dr Rajkumar and M. Foley are PhD Training Fellows at the Medical Research Council (MR/S021108/1 and MR/V001620/1), J.P. Howard is a PhD Training Fellow at the Wellcome Trust (212183/Z/18/Z), and Dr Nowbar is supported by the NIHR Imperial Biomedical Research Centre. Philips Volcano supplied the coronary pressure wires. We acknowledge support of the NIHR Clinical Research Network and the BHF Centre of Research Excellence at Imperial College London.

## Disclosures

Outside of the submitted work, Dr Seligman reports research funding from Amgen, C.M. Cook reports personal fees from Philips, T.R. Keeble has received nonfinancial support from Philips Volcano, A.S.P. Sharp reports personal fees for Medtronic and Philips, Dr Petracoreports speakers honoraria from Philips and consultancy for Amgen, S. Nijjer reports speaker’s honoraria from Philips, Dr Sen reports speaker’s honoraria from Philips, AstraZeneca and Pfizer, Dr Al-Lamee reports speaker’s honoraria from Philips and Menarini pharmaceuticals. The other authors report no Conflicts.

## Supplemental Materials

Online Figure I

Online Tables I and II

## Supplementary Material


