# Supplementary figures and images for: Placebo-Controlled Efficacy of Percutaneous Coronary Intervention for Focal and Diffuse Patterns of Stable Coronary Artery Disease
Source: Circ Cardiovasc Interv. 2021 Aug 3;14(8):e009891. doi: 10.1161/CIRCINTERVENTIONS.120.009891 (PMC8366766; doi:10.1161/CIRCINTERVENTIONS.120.009891)

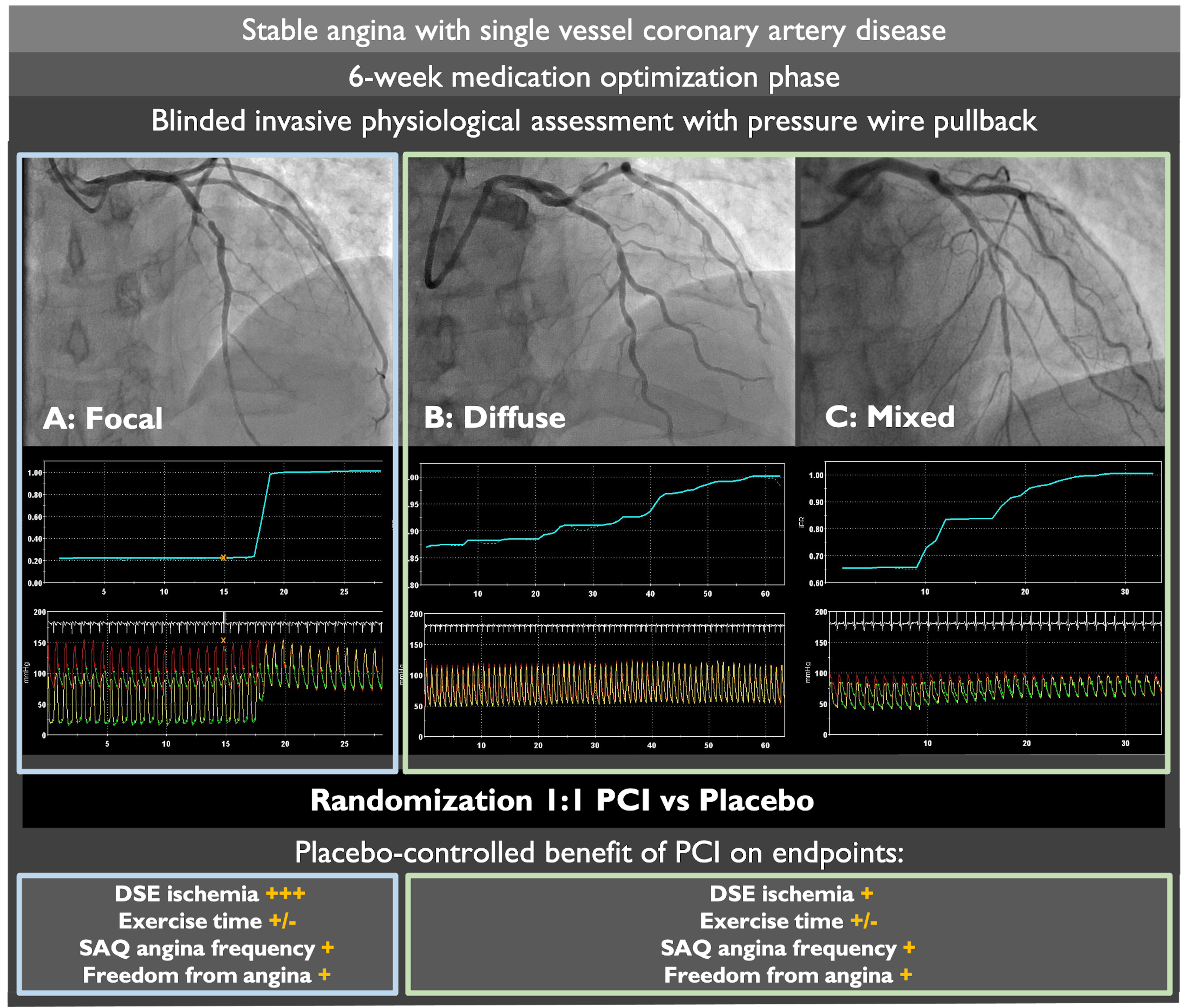

Supplement: Supplementary file 1 [file hcv-14-e009891-s001.tif]
