# Supplementary material for: Placebo-Controlled Efficacy of Percutaneous Coronary Intervention for Focal and Diffuse Patterns of Stable Coronary Artery Disease
Source: Circ Cardiovasc Interv. 2021 Aug 3;14(8):e009891. doi: 10.1161/CIRCINTERVENTIONS.120.009891 (PMC8366766; doi:10.1161/CIRCINTERVENTIONS.120.009891)
Supplement: Supplementary file 2 [file hcv-14-e009891-s002.pdf]

SUPPLEMENTARY APPENDIX

CONTENTS

Page 1

Supplementary Figure I

Effect of physiological pattern of disease on the impact of placebo-controlled PCI on symptom endpoints

Page 2

Supplementary Table I

Sensitivity analysis showing only the results for patients in whom the pre-randomization FFR value was <0.80.

Supplementary Table II

Sensitivity analysis showing only the results for patients in whom the pre-randomization iFR value was <0.89.

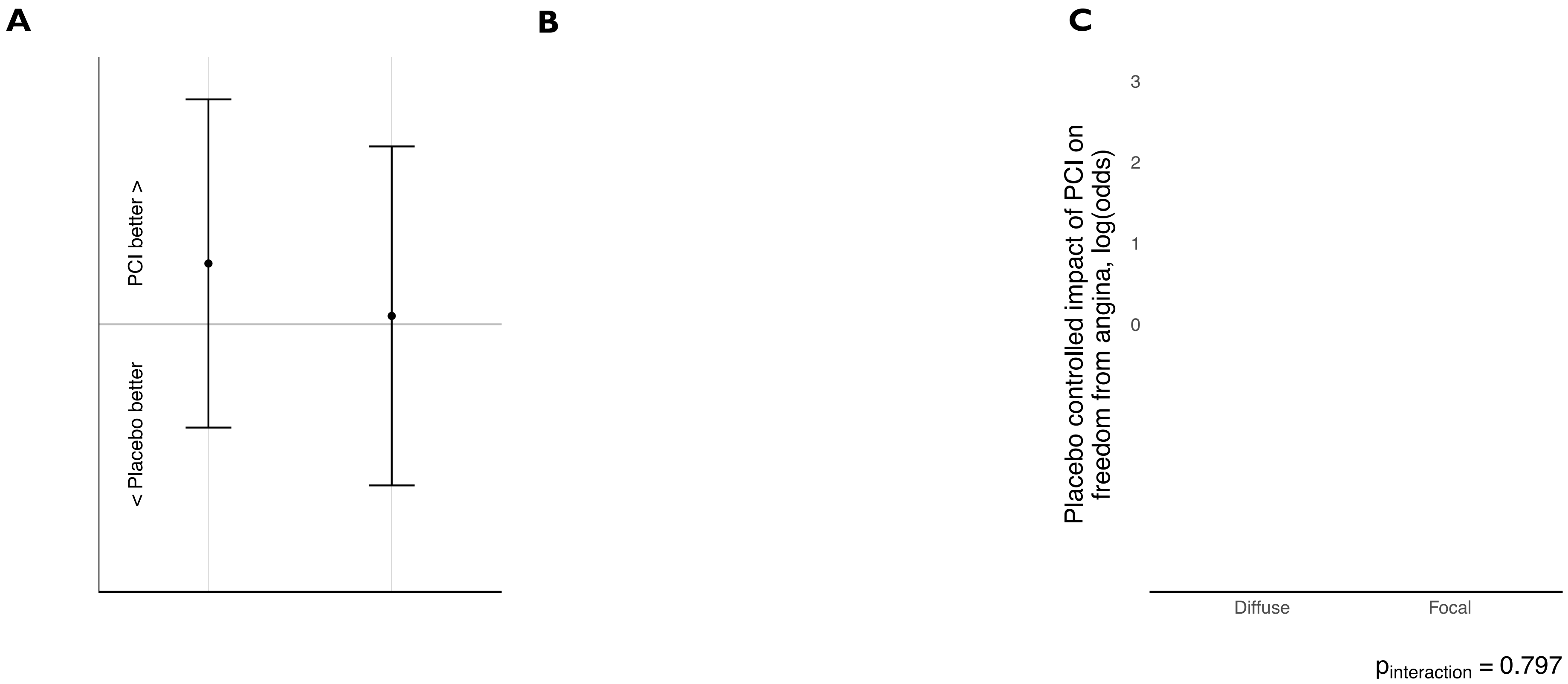

Supplementary Figure I

Effect of physiological pattern of disease on the impact of placebo-controlled PCI on symptom endpoints

The association between physiological pattern of CAD as assessed by iFR-pullback and the benefit of PCI over placebo for (A) exercise time, (B) SAQ angina frequency and (C) SAQ derived freedom from angina, after adjustment for baseline FFR values.

Supplementary Table I

**Entry Criterion**	**PCI (*n*)**	**Placebo (*n*)**	**Endpoint**	**Focal vs Diffuse $P_{interaction}$** Unadjusted for baseline FFR	**Focal vs Diffuse $P_{interaction}$** Adjusted for baseline FFR
FFR ≤0.80	52	43	Stress echo score	0.067	0.076
	62	56	Exercise time	0.619	0.646
	62	56	SAQ Angina Frequency	0.691	0.683

Sensitivity analysis showing only the results for patients in whom the pre-randomization FFR value was <0.80.

Abbreviations: FFR = Fractional flow reserve, PCI = Percutaneous coronary intervention, SAQ = Seattle Angina Questionnaire

Supplementary Table II

**Entry Criterion**	**PCI (*n*)**	**Placebo (*n*)**	**Endpoint**	**Focal vs Diffuse $P_{interaction}$** Unadjusted for baseline iFR	**Focal vs Diffuse $P_{interaction}$** Adjusted for baseline iFR
iFR ≤0.89	46	42	Stress echo score	0.030	0.052
	56	56	Exercise time	0.749	0.703
	56	56	SAQ Angina Frequency	0.545	0.568

Sensitivity analysis showing only the results for patients in whom the pre-randomization iFR value was <0.89.

Abbreviations: iFR = instantaneous wave-free ratio, PCI = Percutaneous coronary intervention, SAQ = Seattle Angina Questionnaire
